# Stiffness-dependent motility and proliferation uncoupled by deletion of CD44

**DOI:** 10.1038/s41598-017-16486-z

**Published:** 2017-11-28

**Authors:** Ziba Razinia, Paola Castagnino, Tina Xu, Alexandra Vázquez-Salgado, Ellen Puré, Richard K. Assoian

**Affiliations:** 1 Department of Systems Pharmacology and Translational Therapeutics, University of Pennsylvania, Philadelphia, PA 19104 USA; 2 Department of Biomedical Sciences, University of Pennsylvania, Philadelphia, PA 19104 USA

## Abstract

Information in the microenvironment guides complex cellular decisions such as whether or not to proliferate and migrate. The effects of soluble extracellular signals on these cellular functions are fairly well understood, but relatively little is known about how the extracellular matrix (ECM), and particularly the mechanical information in the ECM, guides these cellular decisions. Here, we show that CD44, a major receptor for the glycosaminoglycan ECM component hyaluronan, coordinates the motility and proliferative responses to ECM stiffening. We analyzed these cellular responses on fibronectin-coated polyacrylamide hydrogels prepared at a physiologic range of ECM stiffness and found that stiffening of the ECM leads to both cell cycling and cell motility in serum-stimulated primary mouse dermal fibroblasts. Remarkably, deletion of CD44 impaired stiffness-stimulated motility of the primary cells without affecting other hallmark cellular responses to ECM stiffening including cell spread area, stress fiber formation, focal adhesion maturation, and intracellular stiffening. Even stiffness-mediated cell proliferation was unaffected by deletion of CD44. Our results reveal a novel effect of CD44, which is imposed downstream of ECM-mechanosensing and determines if cells couple or uncouple their proliferative and motility responses to ECM stiffness.

## Introduction

Stiffening of the extracellular matrix (ECM) microenvironment is a common feature of many diseased states including breast and pancreatic cancer, cardiovascular diseases, and lung and liver fibrosis^1–7^. Cellular responses to this stiffening have been well studied and generally include concurrent increases in proliferation and motility^2,8–15^, which then contribute to further disease progression^5,6,13,16^. However, cell proliferation and motility may also need to be separated *in vivo*. For example, cells may need to migrate to a particular location and then proliferate at that site during development and the response to injury. This notion raises the possibility that the commonly observed concurrent proliferative and motility responses of cells to increased ECM stiffness can be uncoupled.

Stiffness-mediated changes in proliferation and motility can be investigated *in vitro* using hydrogels coated with ECM proteins such as fibronectin (FN). These hydrogels typically have tunable mechanical properties that can mimic the physiological and pathological elasticity of tissues^17–20^. FN is often used in these platforms as it strongly activates integrin signaling, and its expression pattern *in vivo* is associated with cell proliferation, motility and the response to injury^12,21,22^. Use of these platforms has shown that changes in ECM stiffness regulate cell spreading, organization of the actin cytoskeleton^17,23,24^, the activity of Rho family GTPases^8,12,25^ and focal adhesion formation^8,25,26^ even under constant biochemical conditions.

The information encoded by ECM stiffness is transduced through transmembrane adhesion receptors that bind to ECM proteins such as FN, vitronectin, and collagens. Integrins are the major class of adhesion receptors that connect ECM proteins to the actin cytoskeleton^27,28^. The ECM also includes non-proteinaceous components, particularly the abundant glycosaminoglycan hyaluronan (HA)^29–31^. CD44 is one of HA’s best-studied adhesion receptors^29–31^. Like integrins, CD44 is widely expressed on the surface of vertebrate cells and involved in cell-ECM interactions, adhesion, motility, and proliferation^29^. Both integrins and CD44 lack intrinsic enzymatic activity but associate with a number of signaling enzymes and adapters as well as structural proteins that link to the actin cytoskeleton^27–29,32–35^.

Despite the widespread distribution of CD44, only a few studies have reported on the role of CD44 in mechanobiology^36,37^. Chopra *et al*.^36^ showed that cell spreading and stress fiber formation in response to FN is enhanced if myocytes are cultured on a low-stiffness matrix of thiol-modified HA. Kim and Kumar studied glioblastoma cells on methyacrylated HA hydrogels. They found that CD44 affected stiffness-dependent cell adhesion in the absence and presence of an integrin ligand, but spreading or motility were unaffected when integrin signaling was present^37^. Here, we investigate the role of CD44 in cellular sensing of ECM stiffness. Using FN-coated polyacrylamide hydrogels of varying elasticity, we found that deletion of CD44 inhibited stiffness-dependent motility without comparable effect on cell spread area, focal adhesion abundance and stress fiber formation, or cell proliferation. Our data indicate that CD44 is selectively required for the motility response downstream of ECM stiffness sensing and provide new insight into how cells may couple and uncouple their proliferative and motility responses to ECM stiffening.

## Results

### CD44 is essential for the motility response to ECM stiffness

ECM-coated polyacrylamide hydrogels have been used extensively to study how cells sense and respond to the physical characteristics of their microenvironments because their tunable mechanical properties can mimic the physiological and pathological elasticity of tissues^38,39^. To investigate role of CD44 in stiffness-dependent motility, we generated FN-coated polyacrylamide hydrogels of elastic moduli ranging from 2–4 kPa (hereafter called “soft”) to 18–20 kPa (hereafter called “stiff”). These elastic moduli span the stiffness range that regulates cell cycling and motility in fibroblasts (Supplementary Figure S1).

**Figure 1:**
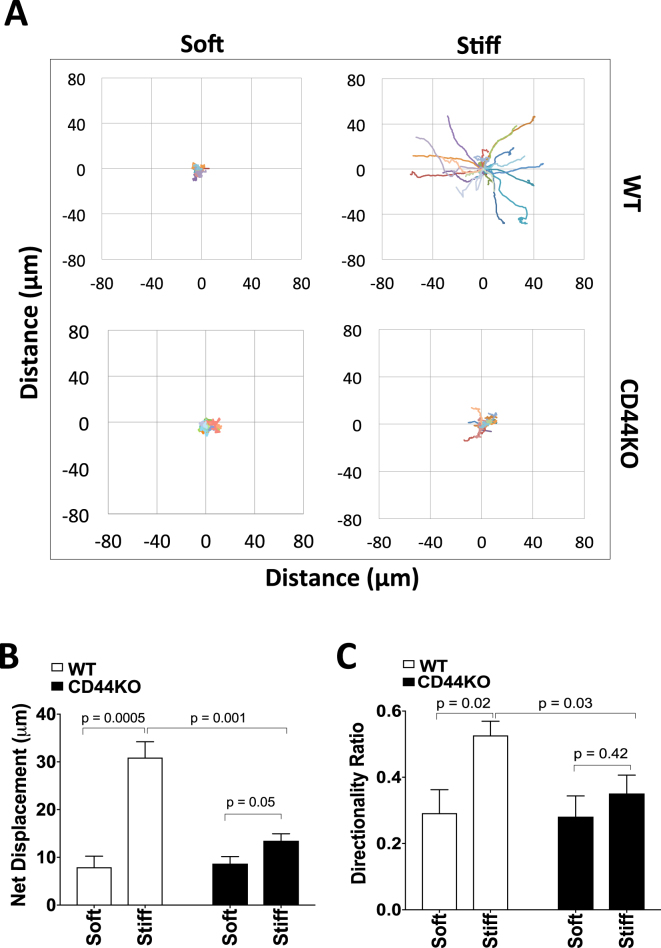
CD44 is essential for the motility response to ECM stiffness. **(A)** WT and CD44KO cells were incubated on soft and stiff FN-coated hydrogels with 10% FBS for 16 h. NucBlue was added to the cells, and the cells were then imaged every 5 min for 5 h. The coordinates of the nucleus centroid using NucBlue were determined, and cell trajectories were plotted. **(B)** Net displacement and **(C)** directionality of WT and CD44KO cells on soft and stiff FN-coated hydrogels were quantified and plotted as mean ± s.e.m. for 5 independent experiments.

Primary dermal fibroblasts isolated from wild-type (WT) and CD44-knockout (CD44KO) mice (Supplementary Figure S2) were plated on the hydrogels, and nuclear tracking in real time was used to examine motility as measured by net displacement, directionality, total distance moved and mean cell speed. Net displacement and directionality increased with increasing stiffness in WT cells; CD44KO cells showed pronounced decreases in net displacement and directionality (Fig. 1A,B), which persisted and increased in significance over the 5 h time period examined (Supplementary Figure S3A,B). These data indicate that CD44 is essential for the normal motility response to ECM stiffness.

The total distance moved and mean cell speed were not statistically different in the primary WT and CD44KO cells on stiff hydrogels though there was a trend towards a lower sensitivity to ECM stiffening in the KO cells (Supplementary Figure 3C,D). On soft hydrogels, both WT and CD44KO cells largely remained in place (Fig. 1A), but real-time imaging showed that they oscillated and therefore have a deceptively high total distance moved and mean cell speed (Supplementary Figure S3C,D). For this reason, we use net displacement as the primary metric to assess stiffness-mediated migratory behavior. Experiments including hydrogels of intermediate stiffness (8–10 kPa) indicate that the motility defect in CD44KO cells is most evident as the ECM was stiffened from 10 kPa to 20 kPa (Supplementary Figure S4).

### CD44 is dispensable for the morphologic response to ECM stiffness

**Figure 2:**
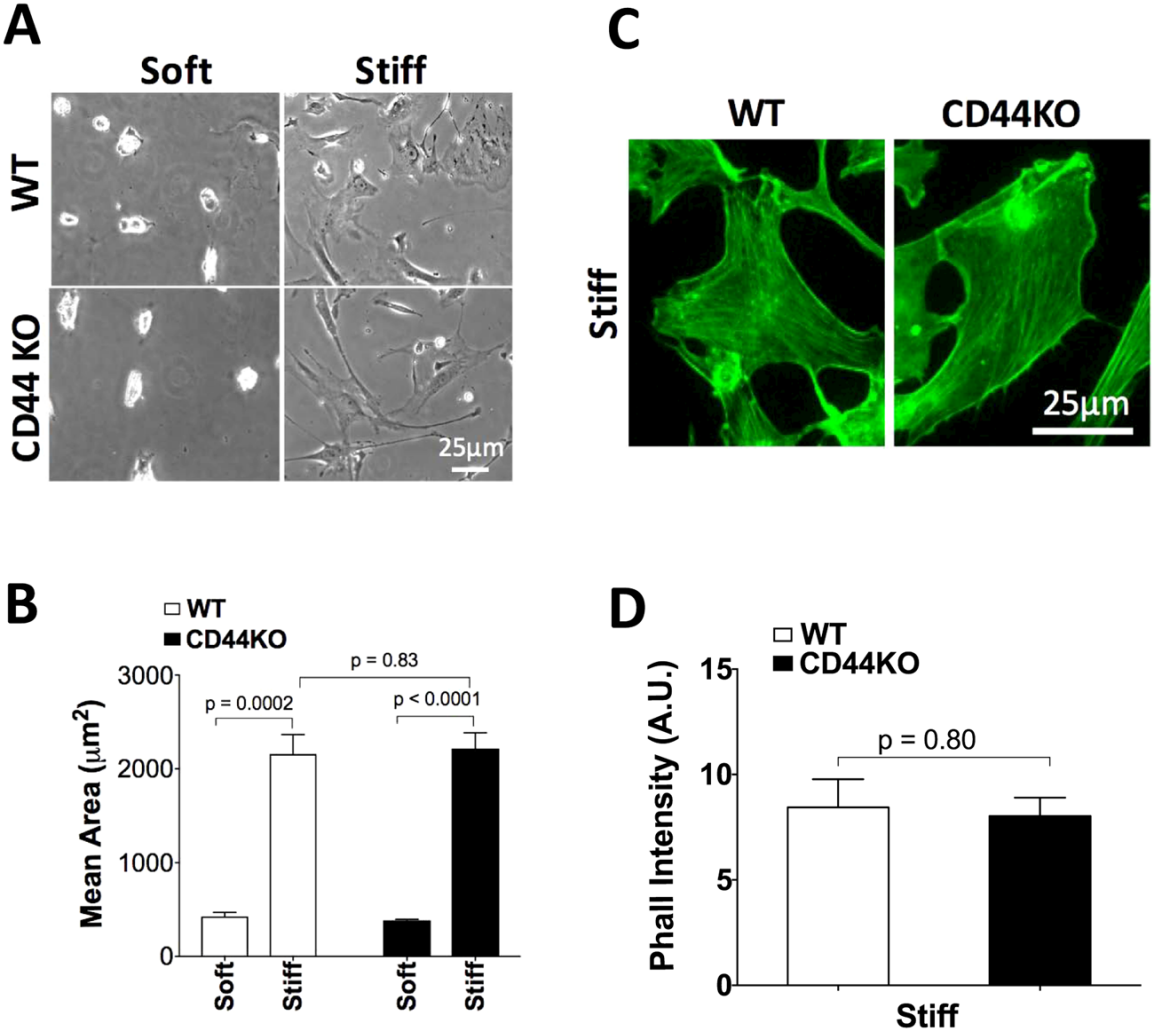
CD44 is dispensable for the morphologic response to ECM stiffness. WT and CD44KO cells were incubated on soft and stiff FN-coated hydrogels with 10% FBS for 16 h. **(A,B)** Cells were fixed and imaged by phase contrast microscopy; cell area was quantified and plotted as mean ± s.e.m. for 4 independent experiments. **(C)** Zoom of region of interest (ROI) of phalloidin-stained images shown in Supplementary Figure S5B. **(D)** Average phalloidin intensity was analyzed and plotted as mean ± s.e.m. for 4 independent experiments.

Cell spreading and f-actin organization are among the earliest responses to ECM stiffening and play pivotal roles in cell motility^13,17,18^. Primary WT and KO cells were plated on stiff and soft FN-coated hydrogels to assess the effect of CD44 on these parameters. Despite the difference in stiffness-dependent motility, both WT and CD44KO cells spread ~4-fold more on stiff as compared to soft substrata (Fig. 2A,B). No difference in spread area (Fig. 2A,B) or cell circularity (Supplementary Figure S5A) was detected in WT and CD44KO cells. Similarly, immunofluorescence analysis of phalloidin-labeled WT and CD44KO cells (Supplementary Figure S5B and Fig. 2C) revealed no difference in stress fiber density (Fig. 2D). Thus, CD44 is not required for cell spreading or the stress fiber response to ECM stiffness.

Focal adhesions connect the ECM to the cytoskeleton through integrins and play a critical role in many cellular processes^40,41^. Formation and maturation of focal adhesions as well as their tyrosine-phosphorylated activation state transduce signals that drive cell migration^40–42^. To determine whether the migratory defect in CD44KO cells is due to changes in focal adhesion maturation and/or activation, we compared the expression and phosphorylation of several important focal adhesion proteins (vinculin, paxillin, FAK and talin) in WT and CD44KO cells after seeding on stiff FN-coated hydrogels.

**Figure 3:**
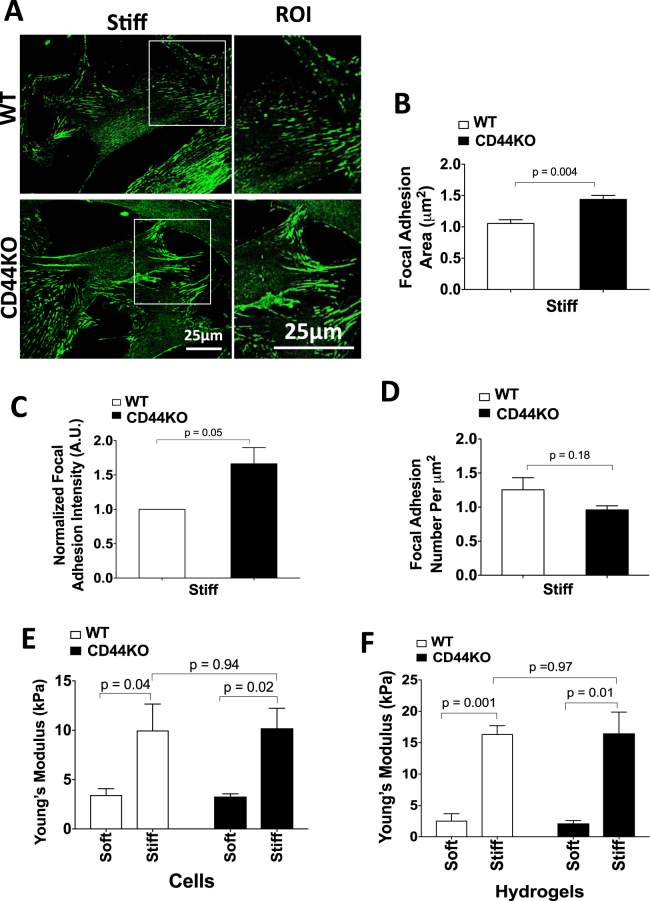
CD44 is dispensable for the mechanotransduction of ECM stiffness into cellular stiffness. (**A)** WT and CD44KO cells were incubated on stiff FN-coated hydrogels with 10% FBS for 16 h. The cells were fixed and stained for paxillin. The zoom shows magnified views of paxillin staining in the region of interest (ROI; white boxes). Focal adhesion area **(B)** normalized intensity **(C)** and number per μm^2^ **(D)** were quantified and plotted as mean ± s.e.m. for 4 independent experiments. **(E**,**F)** WT and CD44KO cells were incubated on soft and stiff FN-coated hydrogels with 10% FBS for 16 h, and the stiffness of the cells **(E)** and hydrogels **(F)** were measured by AFM. Quantification of stiffness was plotted as mean ± s.e.m. for 3 independent experiments.

We examined FAK phosphorylation at Y397 (its autophosphorylation site^41^), paxillin phosphorylation at Y118 (which is involved in focal complex assembly and Rac activation^43^) and vinculin phosphorylation at Y1065 (which promotes f-actin binding and bundling^44,45^). Western blotting and immunostaining for these total and phosphorylated proteins (Supplementary Figures S6–S8) indicated that phosphorylation and abundance of vinculin, vinculin^pY1065^, FAK, FAK^pY397^, paxillin^pY118^ and talin in focal adhesions were similar in WT and CD44KO cells as assessed at both 6 h and 16 h after seeding. Thus, we could not detect an effect of CD44 on focal adhesion maturation and activation in dermal fibroblasts. This conclusion was then confirmed by direct co-staining of WT and CD44KO cells for phosphorylated and total vincuiln (Supplementary Figure S9). We did observe a small increase in area of paxillin-containing focal adhesions (Fig. 3A,B) and a trend towards increased intensity (Fig. 3C) without any significant difference in number of paxillin-containing focal adhesions (Fig. 3D). Atomic force microscopy (AFM) showed that these small changes were insufficient to alter intracellular stiffness (Fig. 3E,F). Collectively, these data indicate that CD44 can strongly regulate stiffness-dependent motility without comparable effect on the formation or activation of focal adhesions or on the mechanotransduction of ECM stiffness into cellular stiffness.

### CD44 is dispensable for stiffness-dependent cell proliferation

**Figure 4:**
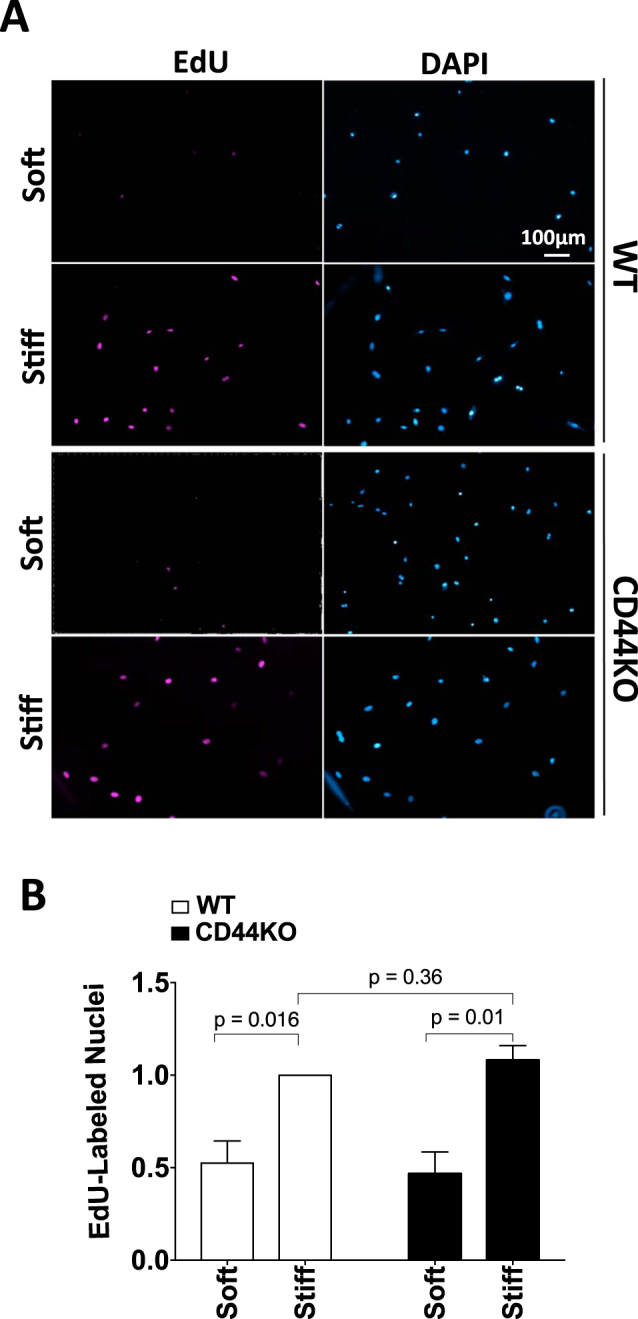
CD44 is dispensable for stiffness-dependent cell proliferation. WT and CD44KO cells were incubated on soft and stiff FN-coated hydrogels with 10% FBS and 10 μM EdU for 16 h. The cells were fixed, stained for EdU-labeled nuclei and counterstained with DAPI (total nuclei). **(A)** Representative images of EdU- and DAPI-labeled nuclei. **(B)** The number of EdU-labeled nuclei was normalized to the WT cells on stiff hydrogels and plotted as mean ± s.e.m. for 3 independent experiments.

The fact that CD44KO cells have an impaired motility response to ECM stiffness, despite a normal morphological response, raised the question of whether CD44 would be required for stiffness-dependent proliferation. To address this possibility, we compared the extent of EdU incorporation in the primary WT and CD44KO cells cultured on hydrogels under the conditions we used for the motility assay. Our initial experiments compared stiffness-sensitive EdU incorporation of subconfluent WT and CD44KO cells. Consistent with our previous reports^8,12^, the number of EdU-labeled nuclei was low when cells were plated on the soft hydrogels and increased when the substratum was stiffened (Fig. 4). The same results were obtained in the absence of CD44 (Fig. 4), showing that CD44 is dispensable for the stiffness-dependent increase in cell cycling.

**Figure 5:**
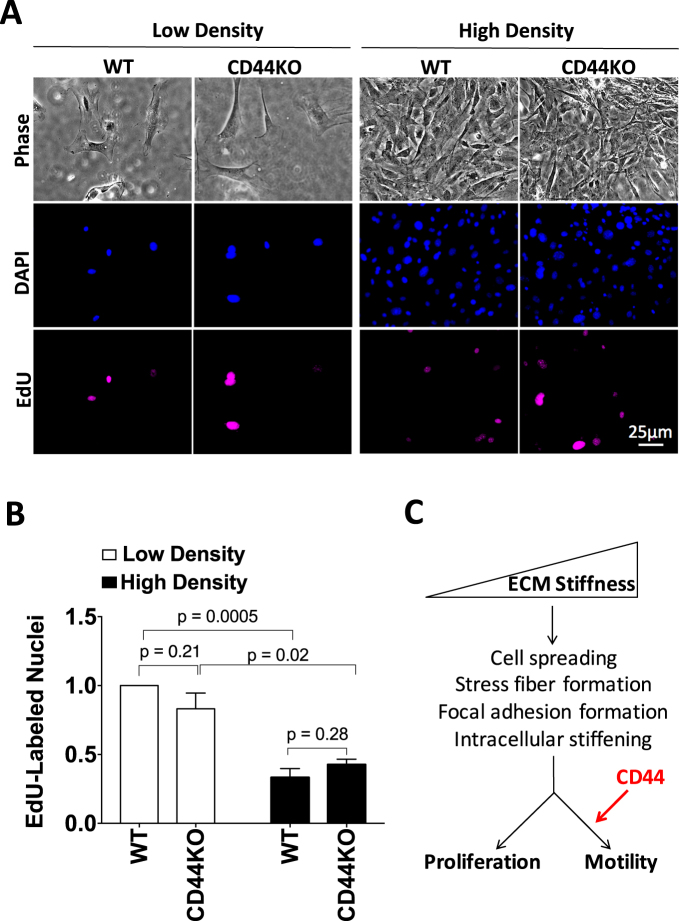
Stiffness-dependent motility requires CD44 but proliferation is regulated by cell density independent of CD44. WT and CD44KO cells were seeded on stiff FN-coated hydrogels at low and high cell density and incubated with 10% FBS and 10 μM EdU for 16 h. **(A)** Representative phase contrast images, DAPI-labeled nuclei, and EdU-labeled nuclei from one of 3 independent experiments. **(B)** Quantified results were normalized to EdU incorporation of the low density WT cells on stiff hydrogels and plotted as mean ± s.e.m. for the 3 independent experiments. **(C)** ECM stiffness stimulates cell spreading, stress fiber and focal adhesion formation and intracellular stiffening. These processes contribute to cell proliferation and motility in response to ECM stiffening. CD44 is selectively required for the motility response to ECM stiffening downstream of these stiffness-dependent events.

Other studies have reported that CD44 inhibits proliferation in high cell density cultures of endothelial cells and dermal fibroblasts^46,47^. To test whether loss of CD44 would regulate stiffness-dependent cell cycling under high-density culture conditions, WT and CD44KO cells were cultured sparsely and densely on stiff FN-coated hydrogels (Fig. 5A,B), and the extent of EdU incorporation was assessed. There was a decrease in EdU incorporation with increasing cell culture density but loss of CD44 did not alter cell cycling in these high-density cultures (Fig. 5A,B). Taken together, our studies indicate that CD44 can selectively affect stiffness-stimulated motility without altering stiffness-stimulated cell proliferation. We also emphasize that our results agree with previous work on plastic (rigid) substrata demonstrating that the proliferative effect of CD44 in fibroblasts requires incubation times beyond those studied here^46^.

## Discussion

While many studies have demonstrated a role for CD44 in cell motility^37,46,48–53^, very little is known about the role of CD44 in stiffness-dependent responses. Here, we show loss of CD44 prevents the normal motility response to ECM stiffening. We considered how CD44 might regulate cell migration in response to stiffness by assessing processes that contribute to cell migration. Surprisingly, we found that loss of CD44 reduced motility without affecting cell spreading, stress fiber formation and focal adhesion maturation and activity when primary dermal fibroblasts were cultured on FN-coated surfaces of physiologic elasticity. Nor did the absence of CD44 affect the transduction of ECM stiffness into intracellular stiffness or stiffness-stimulated EdU incorporation. We therefore posit that our data reveal a new effect of CD44 on cell motility that is imposed downstream of canonical stiffness-mediated mechanosensing and stress fiber formation (Fig. 5C).

Kim and Kumar have also examined the effect of CD44 in regulation of stiffness-dependent motility^37^. In these studies CD44 was knocked-down in glioblastoma cells, and the cells were cultured on hydrogels of methyacrylated HA^37^. In this system, CD44 knockdown inhibited initial stiffness-dependent adhesion when cells were seeded on 2D HA hydrogels with or without the integrin ligand RGD. However, knockdown of CD44 did not strongly affect spreading or motility when the cells were seeded on the RGD-HA hydrogels. Since our studies use hydrogels coated with FN, an RGD-containing integrin ligand, the results we obtained are consistent with those in glioblastoma cells in that integrin-based signaling is likely supporting stiffness-dependent changes in spreading, stress fiber formation, focal adhesion assembly and cell cycling in CD44 KO fibroblasts despite the defect in motility.

How might CD44 regulate motility without affecting stress fiber formation and focal adhesion assembly? Force provided by polymerization of the cortical actin at the leading edge is important for initiation of cell migration as it powers membrane protrusions and drives cell migration^54^. Thus, a stress fiber-independent effect of CD44 on cell motility may be mediated through the cortical actin cytoskeleton. The cortical actin network is in part regulated by ERM proteins, which associate with the cytoplasmic tail of CD44^29,34^. ERM proteins modulate cortical architecture and provide structural links to strengthen the cell cortex by crosslinking actin filaments to the membrane^55^. The ERM-CD44 interaction is important for cell motility, and both CD44 and ERM proteins localize to leading edge lamellipodia and induce front-rear cell polarity^29,48,49,52,53,56,57^.

CD44 may also regulate cell motility through an adhesion-independent mechanism involving CD44 proteolytic cleavage. CD44 undergoes sequential proteolytic cleavage of the extracellular domain (ECD) and intracellular domain (ICD)^58–62^. Cleavage of the ECD is important for CD44-mediated migration^59,63^. The ICD acts as a signal transduction molecule, as it translocates to the nucleus and can mediate gene transcription^58,63,64^. Perhaps this pathway regulates transcription of genes involved in stimulating cell motility downstream of ECM stiffness-sensing and adhesion-dependent signaling.

In an earlier study, we examined the effect of CD44 on cytoskeleton architecture and motility of lung fibroblasts cultured on a rigid (glass and plastic) substrata^48^. This study, which used a scratch assay, also showed a positive effect of CD44 on directionality, but cell velocity increased and stress fibers decreased in CD44KO cells. Differential activation of TGF-β was a key feature of those studies^48^. Future work will be required to determine if the different results in that study and the present report reflect a distinct biology of skin versus lung fibroblasts, or a fundamental differences in signaling when cells are cultured on rigid substrata versus ones of physiological relevant elasticity.

Our work reveals a new link between CD44 and the motility response to ECM stiffness and suggests that CD44 can uncouple the proliferative and motility responses of cells to increased rigidity of the ECM (Fig. 5C). Interestingly, we find that WT and CD44KO cells respond similarly to initial increases in ECM stiffness (from 2–10 kPa), but further increases in ECM stiffness selectively fail to affect CD44KO cells. Thus, the role of CD44 in cell motility *in vivo* will likely depend on the degree to which the ECM stiffens.

## Methods

See supplementary methods for antibodies and reagents and immunofluorescence and western blotting protocols.

### Cell lines and culture conditions

Primary dermal fibroblasts were isolated from 3–4 month male WT and CD44KO mice on a C57BL/6 background and used between passages 1–5. Animal work in this study was carried out in accordance with relevant guidelines and regulations and approved by the Institutional Animal Care and Use Committee of the University of Pennsylvania. The cells were cultured in Dulbecco’s modified Eagle’s medium (DMEM) (Invitrogen) containing 10% fetal bovine serum (FBS), and 50 μg/ml gentamicin. Cells were incubated at 37 °C in a humidified atmosphere containing 10% CO_2_.

### Polyacrylamide hydrogel preparation

Our detailed protocol for preparation of FN-coated polyacrylamide hydrogels has been described previously^65^. Briefly, to covalently attach polyacrylamide gels to glass coverslips (Fisher), “reactive” cover slips were generated by incubation with NaOH followed by addition of 3-APTMS. Glutaraldehyde was used to cross-link the 3-APTMS to the polyacrylamide gel. Solutions containing a constant acrylamide concentration of 7.5%, and bis-acrylamide concentrations of 0.03% for “soft gels” (2–4 kPa), 0.15% for “intermediate stiffness gels” (8–10 kPa) and 0.3% for “stiff gels” (18–20 KPa) were prepared. Ammonium persulfate and TEMED were used to polymerize the hydrogels. N-hydroxysuccinimide (NHS) was incorporated into the solution to crosslink ECM protein to the hydrogel. The hydrogels were then coated overnight by incubation in 0.5 ml of 5 μg/ml FN (Calbiochem) in PBS.

### Random motility assay and quantification

Asynchronous cells were seeded on FN-coated hydrogels in DMEM with 10% FBS and incubated overnight in a 24-well plate to allow cells to fully spread. NucBlue (~50 μl/ml; Thermo Fisher) was added 16 h after seeding and incubated with the cells for 30 min at 37 °C. The plate was transferred to a humidified chamber (5% CO_2_) mounted on a DeltaVision or EVOS microscope. Cells were then imaged by phase contrast microscopy and at 360 nm (to monitor the NucBlue label) every 5 min for 5 h using a 10X objective. Random motility was quantified by nuclear tracking using the Fiji TrackMate Plugin. The coordinates of the nucleus centroid were determined and used to calculate cell trajectories, net displacement (distance from origin), total distance, average cell speed, and directionality using the DiPer open-source computer program^66^. Cells entering or exiting the field of view or dividing during the time-lapse imaging were excluded from the analysis. 20–30 cells were analyzed per condition in each independent experiment.

### Image analysis

To determine the intensity of stress fibers, three lines were drawn across different regions of stress fibers within each cell, and a plot profile (distance along the line vs. pixel intensity) was obtained^67^. Background signal intensities were subtracted by fitting a trend line to the plot profile in Excel. All numbers less than or equal to zero were changed to 0, and a Riemann sum was used to obtain the area under the intensity plot. Finally, the area was normalized to the length of the drawn line, and this area/distance ratio was defined as total actin intensity. 6–10 cells were analyzed per condition in each independent experiment.

The intensity of focal adhesion proteins were quantified with Fiji by measuring raw integrated densities of the signal excluding the perinuclear region. Background raw integrated densities were subtracted, and this net integrated density was then normalized to the total measured cell area. Morphometric analysis of area and number of focal adhesions excluding the perinuclear region was also conducted using Fiji. 6–10 cells were analyzed per condition in each independent experiment.

Cell area and circularity were measured by manually tracing the outline of phase images and quantifying using Fiji. Circularity of 1.0 indicates a perfect circle, and circularity values approaching zero indicate increasingly elongated polygons. 20–25 cells were analyzed per condition in each independent experiment.

### EdU incorporation assay

Asynchronous cells were seeded on FN-coated hydrogels in DMEM with 10% FBS for 1 h before addition of 10 µM 5-ethynyl-2′-deoxyuridine (EdU; Invitrogen). Cells were fixed 16 h after incubation. EdU was visualized using the Click-iT EdU Imaging Kit (Invitrogen) according to the manufacturer’s instructions. Nuclei were stained with DAPI, and hydrogels were mounted onto glass microscope slides. Approximate 4–6 fields of view were manually counted per sample to determine the number of EdU-labeled nuclei relative to DAPI-stained nuclei. 30–50 cells were counted in each of 3 independent experiments.

### Atomic force microscopy

Asynchronous cells were seeded on FN-coated hydrogels, and the media was changed to phenol red-free DMEM with 10% FBS after 16 h. AFM in force mode was used to monitor the intracellular stiffness of single adherent cells on hydrogels using a DAFM-LN or Catalyst Bioscope AFM mounted, respectively, on a Zeiss Axiovert or Nikon Eclipse TE 200 microscope. Cells were indented with a standard silicon nitride cantilever (spring constant = 0.06 N/m) with a 1-μm spherical tip. To quantify the intracellular stiffness (E; Young’s modulus), the first 500 nm of indentation was fit using the Hertz model for a sphere. Three measurements of intracellular stiffness were collected at regions of the cell between the nucleus and the periphery, and ten cells were measured per condition. AFM force curves were analyzed and converted to Young’s modulus using custom MATLAB scripts (generously provided by Paul Janmey, University of Pennsylvania) or NanoScope Analysis software (Bruker). Regions of the hydrogels lacking cells were probed to determine hydrogel stiffness.

### Statistical analysis

Statistical analyses were performed using Prism 6 software (GraphPad Software, Inc). Data are presented as mean ± s.e.m. of the indicated number of independent experiments and were analyzed using an unpaired t-test. *p* values < 0.05 were considered statistically significant.

### Data availability

Primary data used in figures are available on reasonable request.

## Electronic supplementary material

Supplementary Figures and Methods
