# Supplementary material for: Stiffness-dependent motility and proliferation uncoupled by deletion of CD44
Source: Sci Rep. 2017 Nov 28;7:16499. doi: 10.1038/s41598-017-16486-z (PMC5705666; doi:10.1038/s41598-017-16486-z)
Supplement: Supplementary file 1 — Supplementary Figures and Methods [file 41598_2017_16486_MOESM1_ESM.pdf]

SUPPLEMENTARY INFORMATION

Stiffness-dependent proliferation and motility uncoupled by deletion of CD44

Ziba Razinia[1], Paola Castagnino[1], Tina Xu[1], Alexandra Vázquez-Salgado[1], Ellen Puré[2], Richard K. Assoian[1*]

[1]Department of Systems Pharmacology and Translational Therapeutics, University of Pennsylvania, Philadelphia, PA 19104, USA and [2]Department of Biomedical Sciences, University of Pennsylvania, Philadelphia, PA 19104, USA.

*Corresponding author
Address correspondence to Richard K. Assoian at: assoian@upenn.edu

Supplementary Figures S1-S9 and Supplementary Methods

SUPPLEMENTARY FIGURES

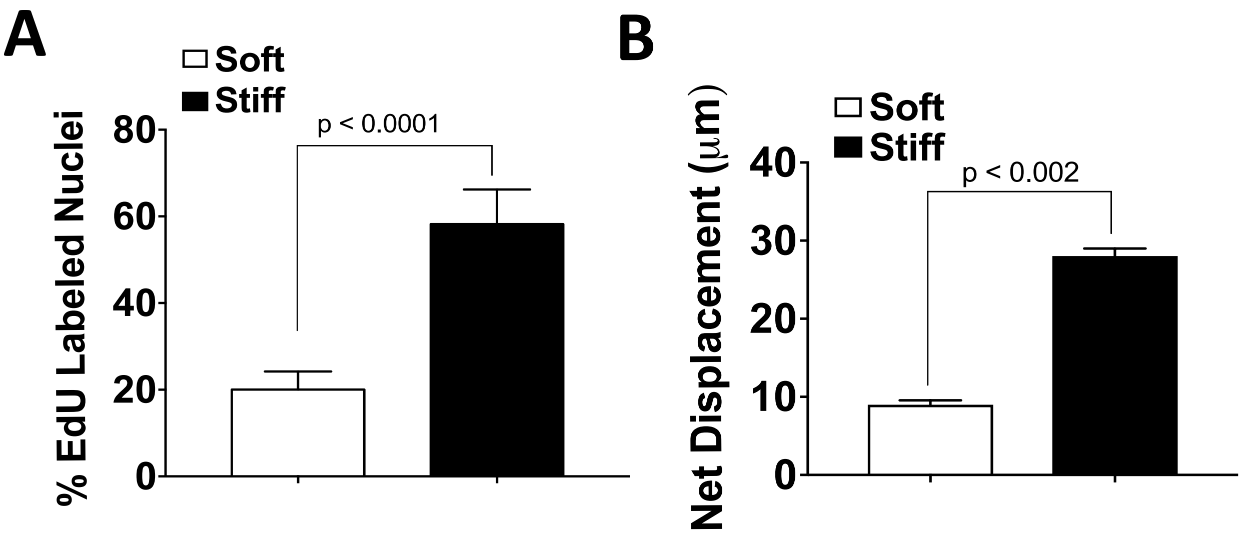

Figure S1. Cell motility and proliferation are regulated by ECM stiffness. (A) Mouse embryonic fibroblasts were seeded on soft and stiff FN-coated hydrogels and incubated with 10% FBS and 10 μM EdU overnight. Cells were then fixed, stained for EdU and counterstained with DAPI. The percent of EdU-labeled nuclei was quantified by manual counting of EdU- and DAPI-labeled nuclei and plotted as mean ± s.e.m. for 5 independent experiments. **(B)** Cells were seeded on soft and stiff FN-coated hydrogels with 10% FBS and incubated overnight. NucBlue was added and cells were imaged every 5 min for 5 h. The coordinates of the nucleus centroid were determined, and net displacement was quantified and plotted as mean ± s.e.m. for 4 independent experiments.

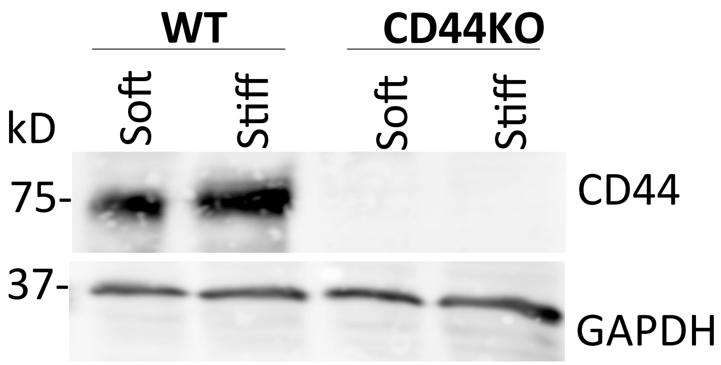

Figure S2. Representative western blot image of WT and CD44KO cells. WT and CD44KO cells were incubated on soft and stiff FN-coated hydrogels with 10% FBS. Cells were lysed in SDS-sample buffer 16 h after seeding and immunoblotted for CD44 using standard western blotting procedures. GAPDH was used as a loading control. Representative image of 4 independent experiments.

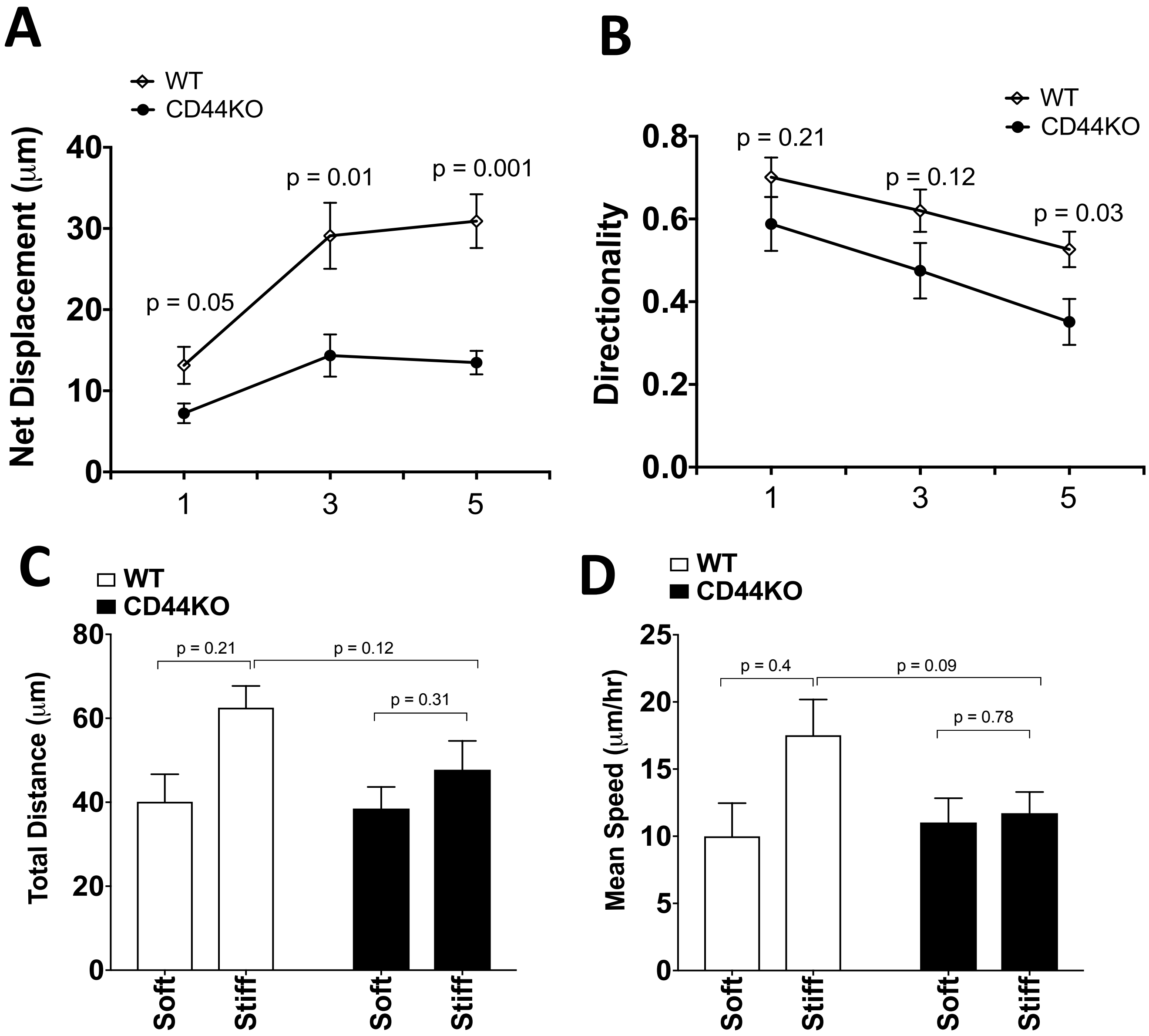

Figure S3. Time course analysis of cell motility and quantification of total distance and mean speed of WT and CD44KO cells. Time course analysis of data in Figure 1 for net displacement **(A)** and directionality **(B)** after 1 h, 3 h and 5 h of imaging. The data in Figure 1 were analyzed for total distance **(C)** and mean speed **(D)** after 5 h of imaging. Results are plotted as mean ± s.e.m. for 3 independent experiments.

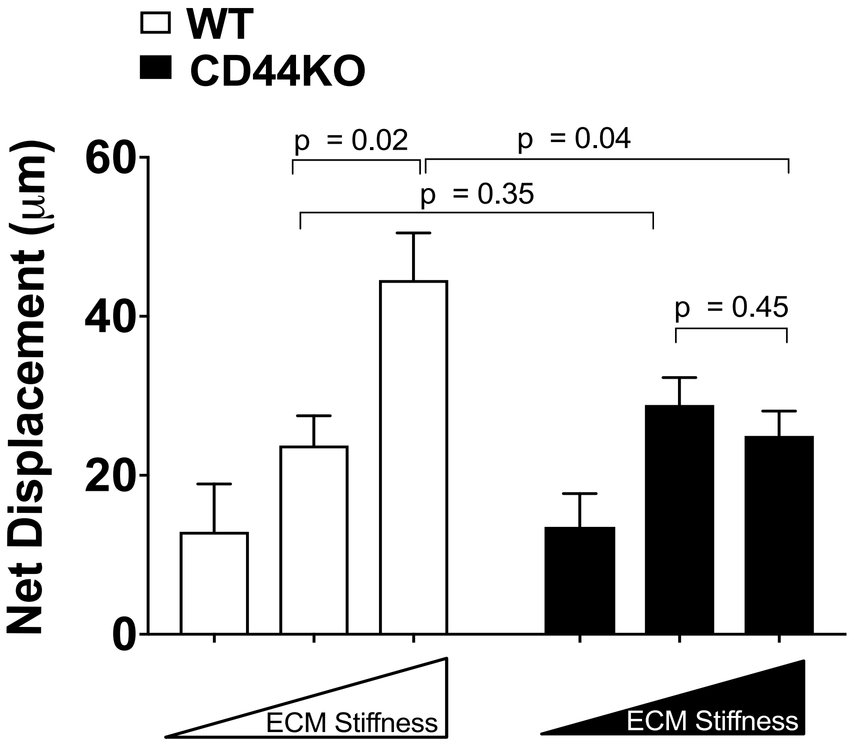

Figure S4. CD44 is not required to sense smaller increases in ECM stiffness. WT and CD44KO cells were incubated on soft (2-4 kPa), intermediate stiffness (8-10 kPa) and stiff (18-20 kPa) FN-coated hydrogels with 10% FBS for 16 h. NucBlue was added and cells were imaged every 5 min for 5 h. The coordinates of the nucleus centroid were determined, and net displacement was quantified and plotted as mean ± s.e.m. for 4 independent experiments.

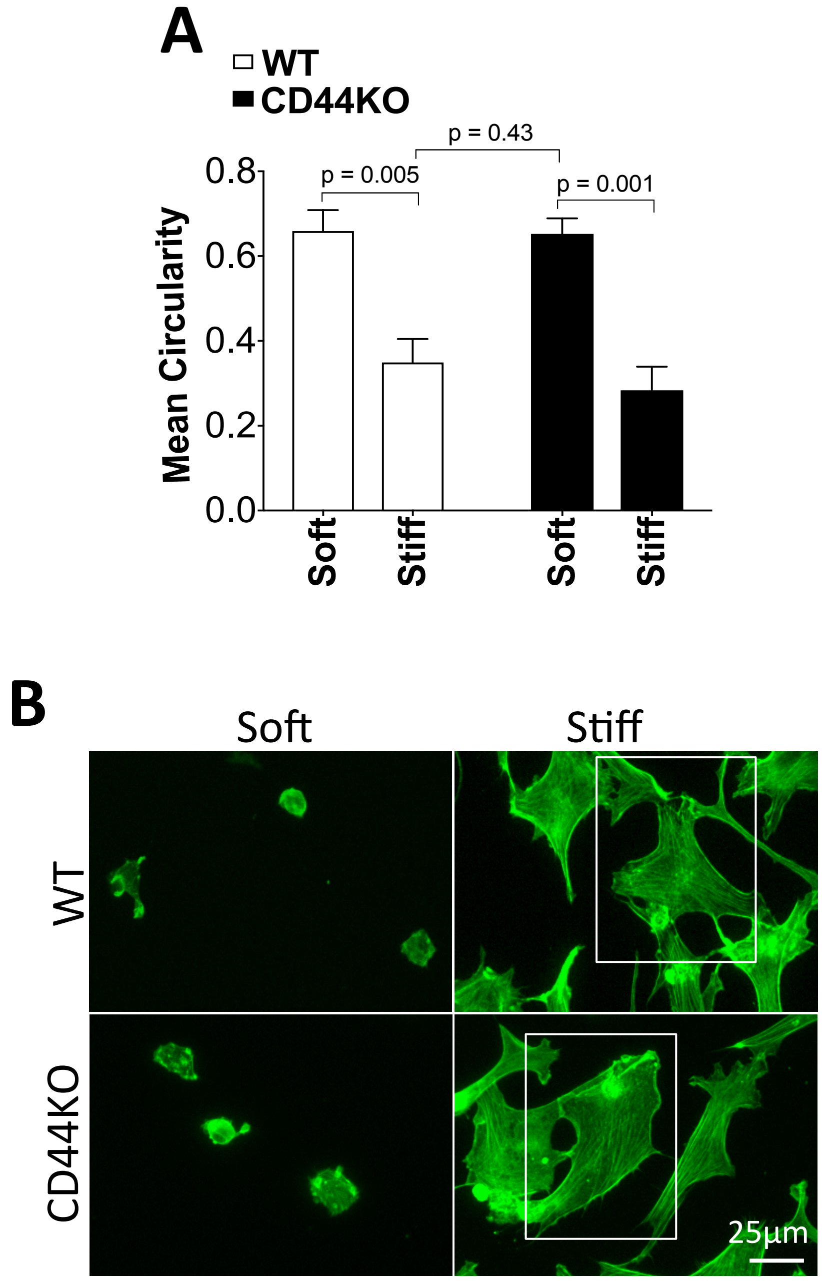

Figure S5. Circularity and phalloidin stain of WT and CD44KO cells.

(A) The data in Figure 2A was analyzed for circularity and plotted as mean ± s.e.m. for 4 independent experiments. **(B)** WT and CD44KO cells were incubated on soft and stiff FN-coated hydrogels with 10% FBS for 16 h. Cells were fixed and stained for phalloidin. Zoomed image of boxed region of interest (ROI) is shown in Figure 2C.

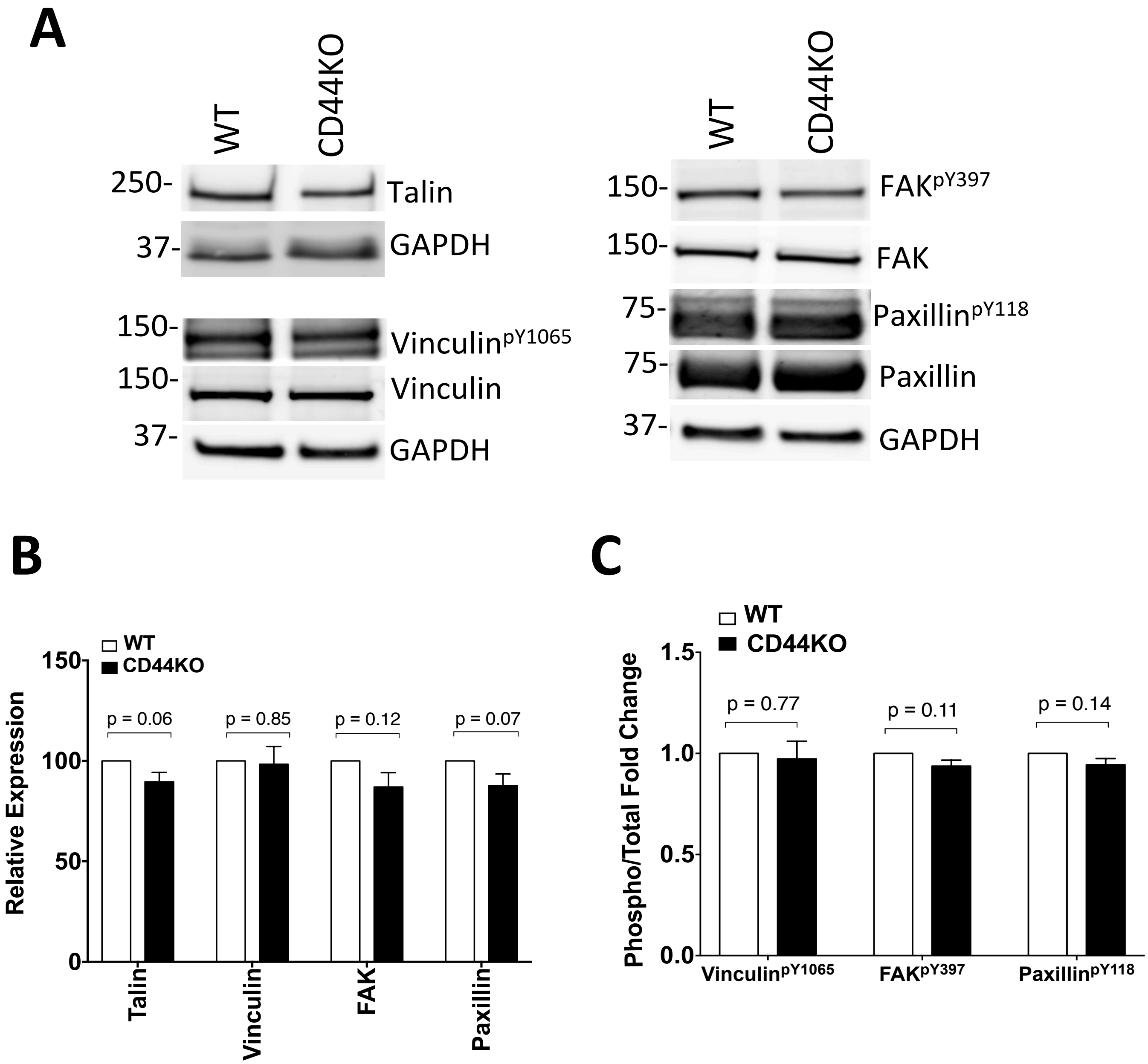

Figure S6. Western blot analysis of focal adhesion proteins in WT and CD44KO cells. WT and CD44KO cells were incubated on stiff FN-coated hydrogels with 10% FBS for 16 h. Cells were lysed in SDS-sample buffer and immunoblotted for talin, vincluin^{pY1065}, vinculin, FAK^{pY397}, FAK, paxillin^{pY118} and paxillin. GAPDH was used as a loading control. **(A)** Representative western blot result from 3 independent experiments. **(B)** Quantification of total talin, vinculin, FAK and paxillin levels relative to GAPDH. **(C)** Quantification of phosphorylated vinculin, FAK and paxillin relative to levels of the respective total protein. Results with CD44KO cells are plotted relative to WT cells and show mean ± s.e.m. for at least 3 independent experiments.

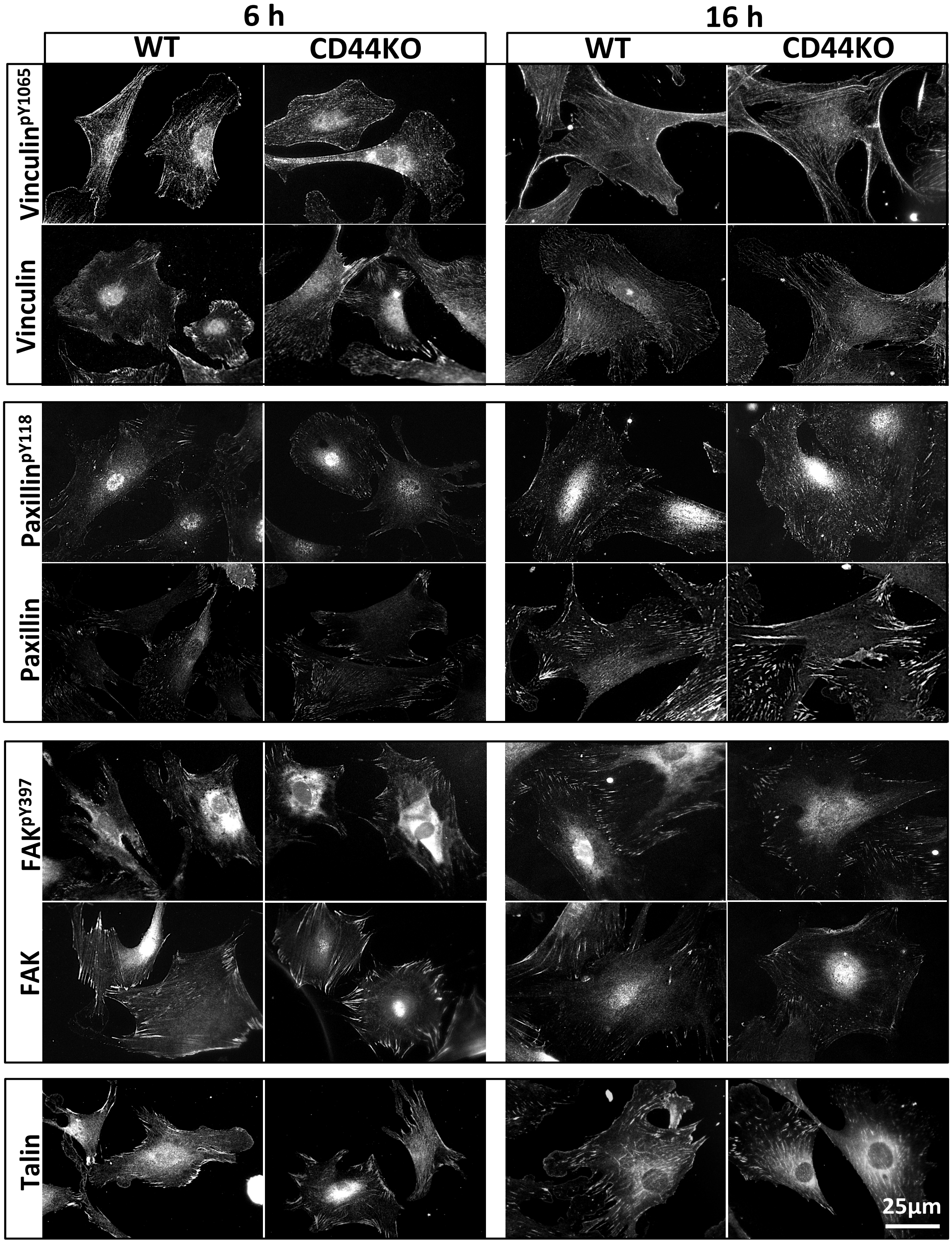

Figure S7. Focal adhesion immunostaining in WT and CD44KO cells. WT and CD44KO cells were incubated on stiff FN-coated hydrogels with 10% FBS and fixed 6 h and 16 h after seeding. Cells were stained for vincluin^{pY1065}, vinculin, paxillin^{pY118}, paxillin, FAK^{pY397}, FAK and talin. The figure shows representative images of 3-5 independent experiments with 50-60 cells assessed for each condition. The paxillin images are replicated in Figure 3A.

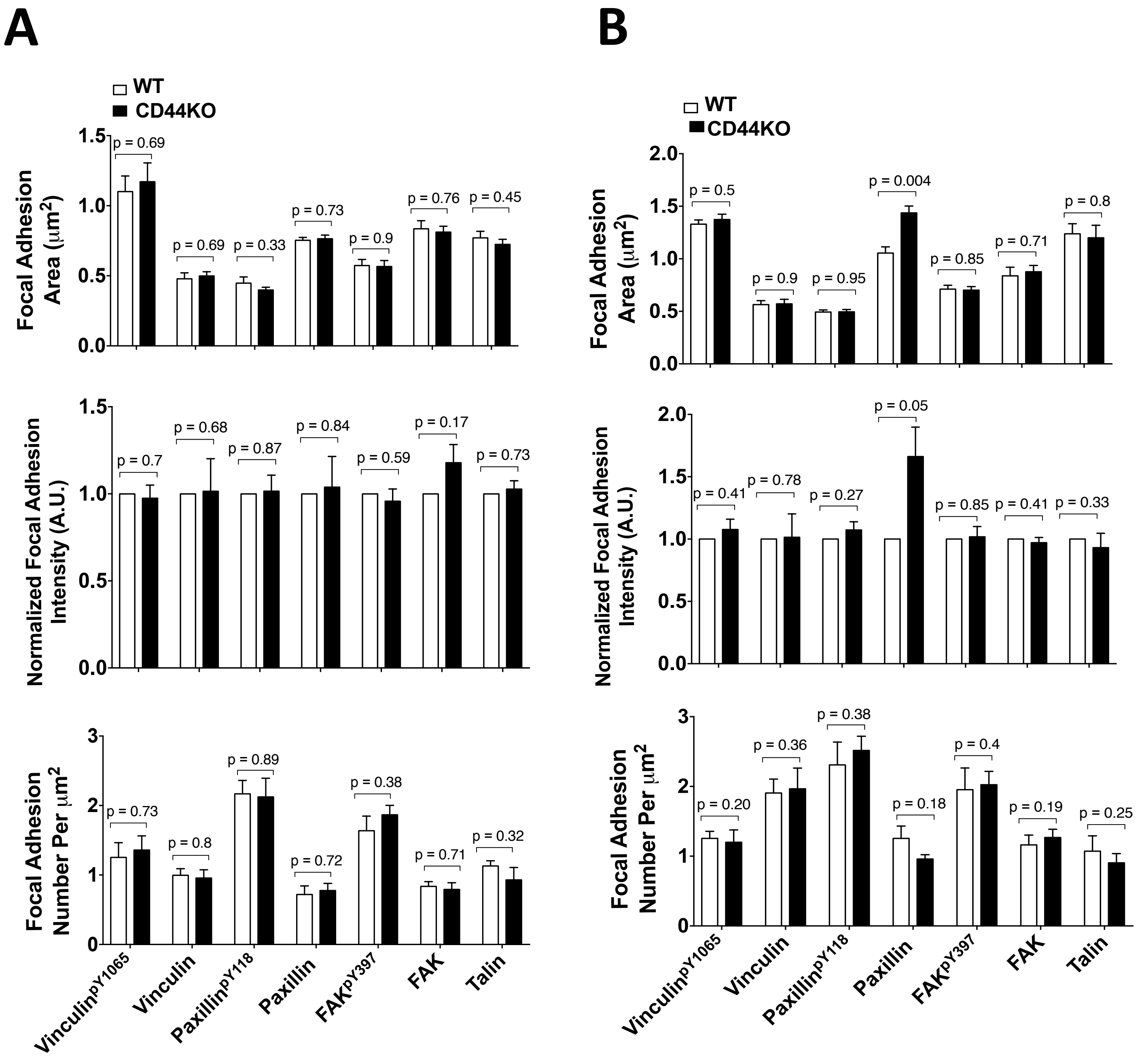

Figure S8. Quantification of focal adhesion immunostaining in WT and CD44KO cells. The experiments from Figure S7 were analyzed as described in Methods and plotted as mean ± s.e.m. for 3 independent experiments at 6 h **(A)** and 16 h **(B)** after seeding. The results for paxillin (16 h after seeding) are replicated in Figures 3B-D.

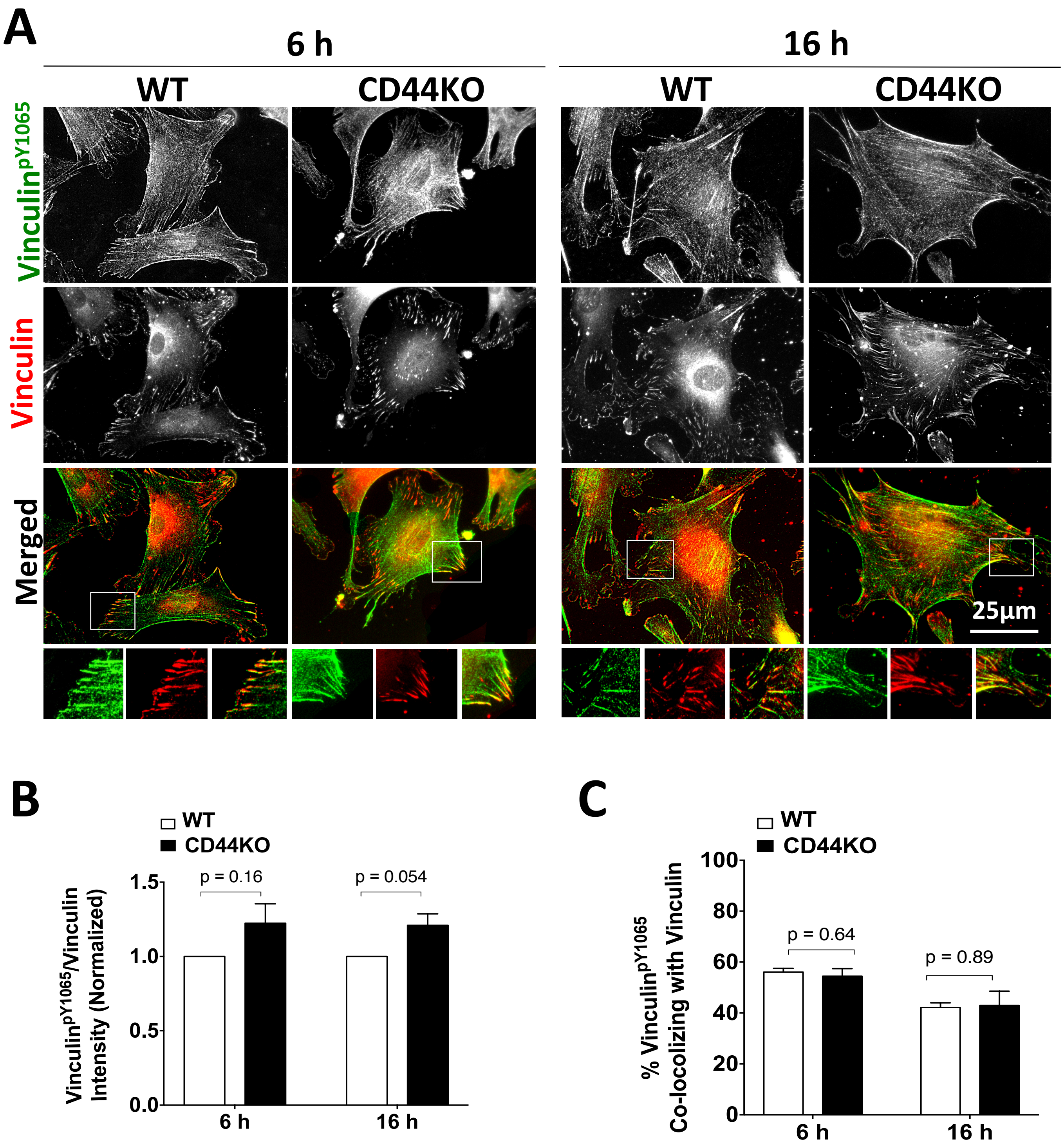

Figure S9. Co-immunostaining analysis of phosphorylated and total vinculin in WT and CD44KO cells. (A) WT and CD44KO cells were incubated on stiff FN-coated hydrogels with 10% FBS and fixed and co-stained for vinculin^{pY1065} and vinculin 6 h and 16 h after seeding. Images were analyzed as described in Supplementary Methods and plotted as mean ± s.e.m. for 3 independent experiments. **(B)** Ratio of vinculin^{pY1065} and vinculin signal intensities. **(C)** Percent vinculin^{pY1065} co-localizing with vinculin.

SUPPLEMENTARY METHODS

Antibodies and reagents

Mouse anti-paxillin (BD Biosciences 610052), mouse anti-phospho-paxillin Y118 (SC-365020), mouse anti-vinculin (Sigma V9131), rabbit anti-phospho-vinculin Y1065 (Thermo Fisher 44-1078G), rabbit anti-FAK (SC-558), rabbit anti-phospho-FAK Y397 (Invitrogen 44-625G) and mouse anti-talin (Sigma T3287) were used for immunofluorescence staining of cells. The same antibodies were used for western blotting with exception of mouse anti-FAK (BD 610088) and rabbit anti-phospho-paxillin Y118 (Thermo Fisher 44-722G). GAPDH (SC-23778) was used as a loading control in the western blots. Rat anti-CD44 was generated in the Puré laboratory. Secondary antibodies for immunofluorescence (AF488 chicken anti-rabbit IgG, AF594 goat anti-rabbit IgG, AF488 donkey anti-mouse IgG, AF594 goat anti-mouse IgG, AF488 donkey anti-rat IgG and AF594 goat anti-rat IgG) were purchased from Thermo Fisher. Phalloidin conjugated with Alexa-488 or Alexa-594 was also purchased from Thermo Fisher.

Immunofluorescence microscopy

Cells plated on FN-coated hydrogels were fixed for 1 h in 3.7% paraformaldehyde in PBS containing 0.05% Triton X-100 and permeabilized for 2 h in PBS containing 0.4% Triton X-100, 2% BSA, and 50 mM NH_4Cl. The coverslips were then rinsed three times in PBS, and incubated with either a 1:100 dilution of fluorophore-conjugated phalloidin overnight at 4°C or a 1:50 dilution of primary antibody at 37°C for 30-40 min followed by overnight incubation at 4°C. For vinculin immunostaining, coverslips were incubated at

37°C for 1 h. Coverslips were then washed three times with 0.3% Triton X-100, 2% BSA in PBS (10 min each wash), and incubated with a 1:100 dilution of secondary antibody for 2 h at room temperature. After washing as above, the hydrogels were mounted using SlowFade Gold Anti-fade mounting agent (Invitrogen). Fluorescent and phase images were acquired on a Nikon Eclipse inverted phase contrast microscope and captured on a QI Click Qimaging camera at 10, 20 or 40X magnification using QCapture Suite Plus software. Images were analyzed using Fiji (http://fiji.sc) as described in Methods section of the main article text.

Western Blotting

Cells were lysed directly in 2X SDS sample buffer. The buffer contained ß-mercaptoethanol except when used to detect CD44. Proteins were resolved by SDS-PAGE and analyzed by western blotting on nitrocellulose membranes blocked with 2% BSA in TBS-T (0.1 M Tris, pH 7.4, 135 mM NaCl, 0.05% Tween 20). The membranes were incubated overnight at 4°C with a 1:500 dilution of primary antibodies, washed in TBS-T, and incubated with a 1:1000 dilution of secondary antibodies. Signals were visualized by enhanced chemiluminescence and detected using an ImageQuant LAS4000 (GE Healthcare). The density of bands was quantified using Fiji.

Quantification of vinculin^{pY1065} and vinculin co-staining

A customized ImageJ plugin to detect and quantify vinculin-containing focal adhesions semi-automatically was developed and kindly provided by Massimiliano Baldassarre (University of Aberdeen). Briefly, maxima points (relative peaks in fluorescence) for vinculin-containing focal adhesions were detected and then an area around these points

was select using a tolerance value. The initial tolerance value was selected based on the pixels standard deviation of the original image and the step was repeated 10 times. A new image with the detected vinculin-containing focal adhesions was then created based on the structures identified in all the iterations. The detected structures in the image were “weighted” for the number of detection times such that focal adhesions identified in all the iterations are brighter than those in one or two iterations. Finally, a manual thresholding step was applied to remove processing artifacts when detected. The thresholded image was then used as a mask to extract vinculin^{pY1065}- and vinculin-containing focal adhesions from the original images. The ratio of vinculin^{pY1065} to vinculin was obtained by dividing the total intensity of vinculin^{pY1065} in vincuilin-containing focal adhesions by the total intensity of vinculin. The percent vinculin^{pY1065} co-localizing with vinculin within the cell periphery was quantified using Fiji’s co-localization plugin. 7-10 cells were analyzed per condition in each independent experiment.
